# A 10-year follow-up study of the association between calcium channel blocker use and the risk of dementia in elderly hypertensive patients

**DOI:** 10.1097/MD.0000000000004593

**Published:** 2016-08-12

**Authors:** Chia-Liang Wu, Shu-Hui Wen

**Affiliations:** a Department of Psychiatry, Taipei Veterans General Hospital, Yuli Branch; b Department of Public Health, College of Medicine, Tzu Chi University, Hualien, Taiwan.

**Keywords:** calcium channel blocker, cohort study, dementia, hazard ratio, propensity score

## Abstract

Calcium channel blockers (CCBs) are widely used for reducing blood pressure of hypertensive patients. Recent reports document the beneficial effects of CCB for preventing dementia; however, the results are controversial. We aim to evaluate the risk of developing dementia among elderly hypertensive patients treated with CCB.

We designed a retrospective population-based cohort study using the records of the National Health Insurance Research Database of Taiwan dated from 2000 to 2010. The study cohort comprised 82,107 hypertensive patients of more than 60 years of age, and 4004 propensity score (PS)-matched pairs were selected according to age, sex, year of hypertension diagnosis, and baseline comorbidities. We employed a robust Cox proportional hazard model to estimate the hazard ratio (HR) of developing dementia in the PS-matched cohort.

The annual incidence of dementia in the CCB-exposure group was significantly lower than that in the comparator group (3.9 vs 6.9 per 1000 person-years, *P* < 0.01) during the follow-up period (4.4 ± 2.5 years). Based on the PS-matched cohort, the adjusted HR of dementia in the CCB-exposure group was significantly lower than that in comparator group (HR = 0.53, 95% confidence interval: 0.39–0.72, *P* < 0.01). Sensitivity and subgroup analyses also confirmed similar findings.

Our results provided evidence for an association between CCB use and a lower risk of developing dementia among the elderly hypertensive patients. Further studies are required to explore the causal relationship between CCB use and dementia.

## 1 Introduction

Calcium channel blockers (CCBs) are commonly recommended as first-line antihypertensives that reduce blood pressure (BP), especially for hypertensive patients >60 years. ^[1]^ CCB comprise drugs that disrupt the transport of calcium through calcium channels and reduce BP by acting on vascular smooth muscles to increase arterial diameter. Free intracellular calcium is an important messenger for many signal transduction pathways of neurons. The maintenance of neuronal viability and function requires the maintenance of intracellular calcium homoeostasis.[ ^2^ ^3^]

The control of the intracellular calcium concentration is impaired during aging, potentially leading to neuronal dysfunction. ^[4]^ Further, amyloid-beta peptide accumulation contributes to the pathogenesis of Alzheimer disease (AD), which makes up 50% to 70% of dementia cases, and evidence indicates that the accumulation of amyloid-beta induces the influx of extracellular calcium in patients with AD. ^[5]^ Neuropathology and cell death may occur due to changes in calcium flux across different cellular membranes, ^[6]^ and it is suggested that CCBs exert a neuroprotection effect ^[7]^ and decrease amyloid-beta accumulation by inhibiting platelet activation in vitro.[ ^8^ ^9^] Therefore, CCBs may have beneficial effect for AD prevention. Besides, vascular dementia, which makes up 25% of dementia cases, is caused by cerebral hypoperfusion and may benefit from the calcium channel blockade, which could improve cerebrovascular perfusion and relaxation of the cerebral vasculature. ^[10]^

The double-blind placebo-controlled Systolic Hypertension in Europe trial found that CCB-based treatments were associated with a lower incidence of dementia (reduced incidence of 7.7–3.8 cases per 10 ^[3]^ patient-years) among elderly people with isolated systolic hypertension. ^[11]^ Further, a cross-sectional study of 1241 hypertensive subjects with the complaints of memory impairment found that CCBs were associated with the decreased risk of cognitive impairment and AD. ^[12]^ In addition, epidemiological studies suggested that the administration of CCB was associated with decelerated cognitive function decline.[ ^13^ ^14^] A Cochrane review examined 14 randomized, placebo-controlled and double-blind trials and concluded that treatment with the CCB (nimodipine) was beneficial for patients with AD. ^[15]^

However, the results of studies concerning the use of CCB to reduce the risk of dementia were not consistent. For example, the Baltimore Longitudinal Study of Aging ^[3]^ recruited 1092 healthy subjects over 60 years of age and found that subjects having treatment with CCB did not reduce the risk of AD (relative risk = 0.3, 95% confidence interval [CI]: 0.07–1.25). The Cache County Study cohort study of 3297 hypertensive subjects claimed that CCB did not reduce the incidence of AD. ^[16]^ A systematic review concluded that it was unclear whether CCB reduced the risk of AD (overall risk ratio is 0.79, 95% CI: 0.53–1.17) in the elderly. ^[17]^ It is worth noting that the definition of the comparator group or CCB exposure varied greatly among different studies.[ ^3^ ^11^ ^12^ ^16^] But a systematic review in 2015 concluded that CCB may be beneficial for preventing AD based on the findings of longitudinal studies, randomized controlled trials, and meta-analyses. ^[18]^ Because hypertensive patients are commonly treated with CCB in Taiwan, the potential beneficial effect of treating the elderly with CCB is worth evaluating. The aim of the present study was to conduct a large population-based cohort study of the residents in Taiwan to determine whether the risk of dementia, including AD or vascular dementia, is reduced in elderly hypertensive patients receiving CCB therapy.

## 2 Methods

### 2.1 Data source

The universal National Health Insurance (NHI) program, which was instituted in Taiwan in 1995, is a single-payer compulsory social insurance plan that covers all types of healthcare institutions and enrolls approximately 99% of the population of Taiwan. The NHI program database contains the registration files and original claims of inpatients and ambulatory patients. To provide access to this database for research purposes, the Ministry of Health and Welfare cooperates with the Bureau of NHI to establish and maintain the NHI Research Database (NHIRD). NHIRD was used for a high-quality epidemiological study ^[19]^ and had a good validity. ^[20]^

We used the Longitudinal Health Insurance Database (LHID), which includes 2000,000 beneficiaries randomly sampled from the Registry of NHIRD (provided by the Health and Welfare Statistics Application Center, Ministry of Health and Welfare) dated between January 1, 2000 and December 31, 2010. LHID is a cohort dataset of original medical claims data that uses a systematic sampling method. There was no significant difference in the distributions of age and sex between the individuals in LHID and all enrollees. ^[21]^ Further, each patient's original identification LHID number is encrypted to protect privacy. We extracted drug information on the basis of the records of dispensations at hospitals, clinics, and contracted pharmacies and identified disease diagnosis from the records of inpatients and ambulatory patients according to the *International Classification of Disease-Clinical Modification*, 9th revision (ICD-9-CM). The Research Ethics Committee of Buddhist Tzu Chi General Hospital, Hualien, approved this study.

### 2.2 Study population

We conducted a retrospective population-based cohort study. New-onset elderly hypertensive patients were eligible for inclusion in the study. First, we extracted hypertensive patients from the 2000,000 records of LHID. The diagnosis of hypertension (ICD-9-CM: 401.x–404.x) was confirmed at least twice from January 1, 2000 to December 31, 2009 and patients who ever been prescribed antihypertensive medications (n = 389,804). The date of the initial diagnosis of hypertension was used as the date of cohort enrollment. Subjects with hypertension diagnosis from January 1, 2000 to December 31, 2000 were excluded to ensure the majority of those included were new-onset patients. Next, patients with history of human immunodeficiency virus infection or thyroid disease before the enrollment were also excluded because they are associated with dementia. We finally included 82,107 patients aged 60 years and older as the study cohort. Taking into account exposure status of CCB use, patients who did not meet the definition of either the CCB group or comparator group were excluded as described below.

### 2.3 CCB-exposure and comparator groups

We identified antihypertensives prescribed during ambulatory visits and the contracted pharmacies from the entry date to 3 months before the end date of follow-up (i.e., exposure-risk period) because the drug effects of CCB with dementia may take at least several months to appear after initial use. We used the anatomical therapeutic code (ATC code) ^[22]^ to classify antihypertensives into 6 classes as follows: CCB (ATC codes C08CA, C08DA, and C08DB), angiotensin receptor blockers (ARBs and ATC code C09CA), angiotensin-converting enzyme inhibitors (ACEIs and ATC code C09AA), diuretics (ATC code C03), α-blockers (ATC code C02CA), and β-blockers (ATC codes C07AA and C07AB). The treatment duration and defined daily dose (DDD) were used to estimate the cumulative exposure to antihypertensives. DDD is a unit to measure prescribed amount of drug and represents the average maintenance dose per day of a drug used for its main indication in adults. We identified all antihypertensive drugs that had a DDD as defined by the World Health Organization. ^[22]^ Number of DDDs was calculated as the total amount of drugs per prescription divided by amount of drug in a DDD. The cumulative DDD (cDDD) of each class of antihypertensive was calculated as the accumulation of DDD during the exposure-risk period.

Among 82,107 subjects, we defined the CCB-exposure group (n = 12,174) as patients treated with a cDDD of CCB >90 and other antihypertensives with a cDDD < 90 during the exposure-risk period. The comparator group (n = 4782) comprised patients treated without any use of CCB and with other antihypertensives with a cDDD < 90. To avoid confounding by indication, we recruited relatively homogeneous hypertensive patients for comparison. Most of hypertensive patients were excluded if they took at least 2 types of antihypertensives with cDDD > 90 during follow-up period (about 40%). In addition, to balance confounding factors between the 2 groups and to reduce bias, we applied propensity score (PS) matching at a ratio of 1:1 for CCB exposure to the matched comparator group. PS, the predicted probability of CCB exposure, was calculated using logistic regression on the basis of patients’ demographics (age and sex), the year of hypertension diagnosis, and baseline comorbidities at enrollment. Cardiovascular diseases (ICD-9-CM code: 410.xx–414.xx), diabetes mellitus (ICD-9-CM code: 249.xx, 250.xx), hyperlipidemia (ICD-9-CM code: 272.xx), chronic renal failure (ICD-9-CM code: 585.xx), heart failure (ICD-9-CM code: 428.xx), arrhythmia (ICD-9-CM code: 427.3x), gout (ICD-9-CM code: 274.xx), benign prostate hypertrophy (ICD-9-CM code: 600.xx), and asthma (ICD-9-CM code: 493) were considered as baseline comorbidities. These diseases were determined by at least 2 outpatient visits 1 year before the enrollment. Then, pairs of CCB-exposure and comparator group were matched on the logit of the PS based on calipers with 0.2 of the standard deviation of the logit of the PS. Furthermore, we might encounter the immortal time bias in this setting. The period from enrollment to the date of CCB treatment started is immortal time (in years). To prevent the immortal time bias, the index date for the start of follow-up was defined as the first prescription date of CCB treatment. For their respective matched comparator, the index date was set to be that of their matched individual with CCB use. Finally, a total of 4004 pairs of PS-matched CCB-exposure and comparator groups were identified.

**Figure 1:**
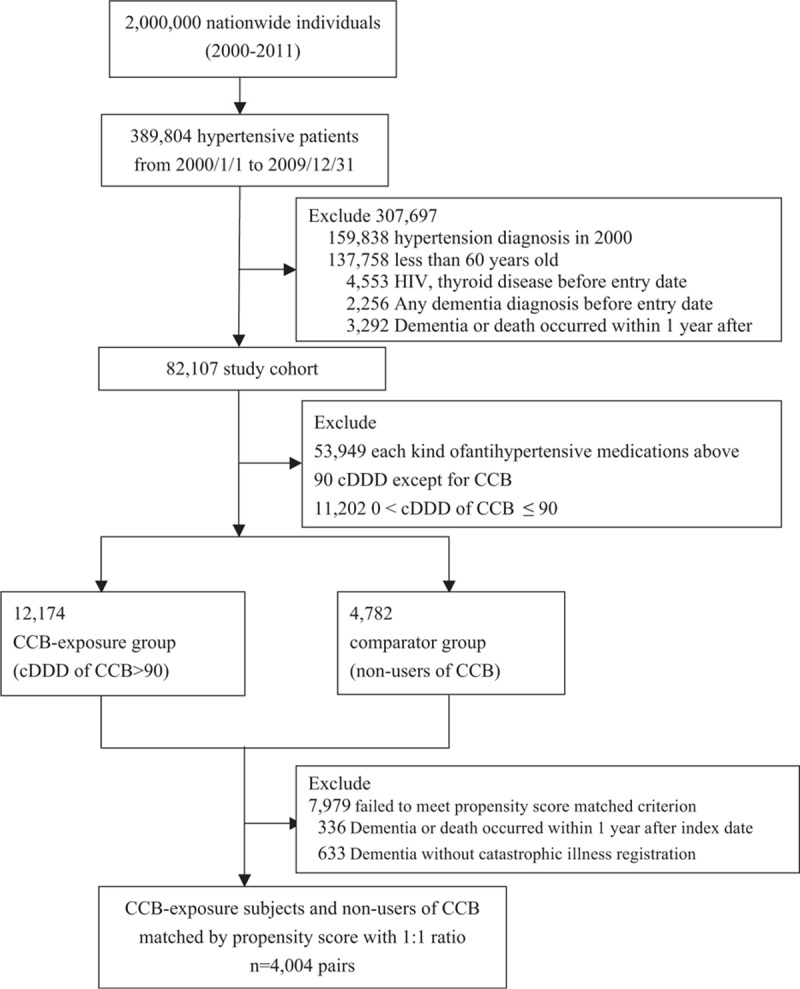
Flowchart of study sample selected. AD = Alzheimer disease, CCB = calcium channel blocker, DDD = defined daily dose, HIV = human immunodeficiency virus.

In order to identify dementia patients with sufficient accuracy, we determined dementia patients as having the primary diagnosis of dementia diagnosis (ICD-9-CM code: 290.0, 290.1, 290.2, 290.3, 290.4, 290.8, 290.9, and 331.0) along with catastrophic illness registration when they had outpatient or inpatient visits from the index date to the end of the study (December 31, 2010). In Taiwan, a board-qualified psychiatrist or neurologist primarily confirmed the diagnosis of dementia based on the diagnostic criteria of the Diagnostic and Statistical Manual of Mental Disorders, Fourth Edition. In addition, patients suspected of dementia were further assessed by medical history, activities of daily living, physical condition, behavior, social function, cognitive function, blood tests, and brain imaging to determine the cause of dementia. Furthermore, dementia patients were eligible for having catastrophic illness certificates which could exempt patients from co-payment. The end date of follow-up was defined as the date of dementia diagnosis, loss to follow-up (e.g., death), or December 31, 2010, whichever occurred first. Further, patients were excluded if the time of follow-up was <1 year and those with dementia before the index date. Figure 1 presents a flowchart describing patient selection. Our study also included the utilization of medical resources such as annual times of ambulatory visits and hospitalizations as well as the dose of other antihypertensives during follow-up.

### 2.4 Statistical analysis

Categorical variables were presented as numbers and percentages, and continuous variables were presented as the mean and standard deviation. We compared the CCB-exposure group with the comparator group using an independent sample *t* test for continuous variables and the chi-square test for categorical variables. In addition, standardized mean difference (SMD) which was calculated as the difference of means for the 2 groups divided by the pooled standard deviation for unmatched and PS-matched cohort. The absolute SMD less than 0.1 indicated good balance. For PS-matched data, Cox proportional hazard models with a robust estimator ^[23]^ (termed “Robust Cox model” hereafter) were adopted to obtain a precise estimate of the standard errors of regression coefficients. The Robust Cox model was designed to account for the dependence of matched pairs. Cox proportional hazard models were used to estimate the hazard ratio (HR) of dementia associated with CCB use and the 95% CI, both using CCB-exposure-only and full adjustment for covariates. The multivariate Cox model was adjusted for potential confounding factors such as age, sex, comorbidities, the year of hypertension diagnosis, annual ambulatory visit times, annual hospitalized times, and cDDDs of other antihypertensives. A proportional hazards assumption was used to validate the application of Cox proportional hazard models. Further, PS was used as a covariate via Cox-regression adjustment. A 2-tailed *P* = 0.05 was considered statistically significant. We performed sensitivity analyses to evaluate the risk of dementia at varying cDDD thresholds of drug dose (120, 150, and 180 cDDD) for CCB exposure, and we defined the exposure period to CCB use based on the drug supply days (90, 120, 150, and 180 days). In addition, we carried out subgroup analyses based on baseline characteristics, including gender, age, comorbidities, and the year of hypertension diagnosis. All analyses were conducted using SAS software (version 9.2; SAS Institute Inc., Cary, NC).

## 3 Results

The mean age of the 16,956 eligible participants was 70.3 years, and 51.7% were female. The most common comorbidities were hyperlipidemia (14.3%), followed by diabetes mellitus (12.0%) and cardiovascular disease (10.3%). Age, sex, and baseline comorbidities (diabetes mellitus, hyperlipidemia, depression, heart failure, benign prostate hypertrophy, and asthma) significantly differed between the CCB-exposure and comparator groups (Table 1). In the PS-matched cohort (Table 2), their demographics and baseline comorbidities were comparable. The absolute SMD of all baseline variables was <0.1, indicating good balance after PS matching. During follow-up period, the cDDDs of other antihypertensive medications were significantly different for CCB (152.7 vs 0, *P* < 0.01), ACEI (3.0 vs 3.4, *P* = 0.046), and α-blocker (1.0 vs 1.5, *P* < 0.01). However, the statistically significant mean differences in the cumulative doses of ACEI and α-blocker may not be clinically significant because of the large sample size. Total immortal time was 4004 years of follow-up, which accounted for 18.9% of the total person-years of follow-up in CCB-exposure group. After excluding immortal time, the overall incidence of dementia (n = 191) was 5.5 cases per 10^3^ person-years during a mean follow-up of 4.4 ± 2.5 years. The incidence of developing dementia in the CCB-exposure group was significantly lower than that in the matched comparator group (3.9 vs 6.9 per 10^3^ person-years, *P* < 0.01).

**Table 1:**
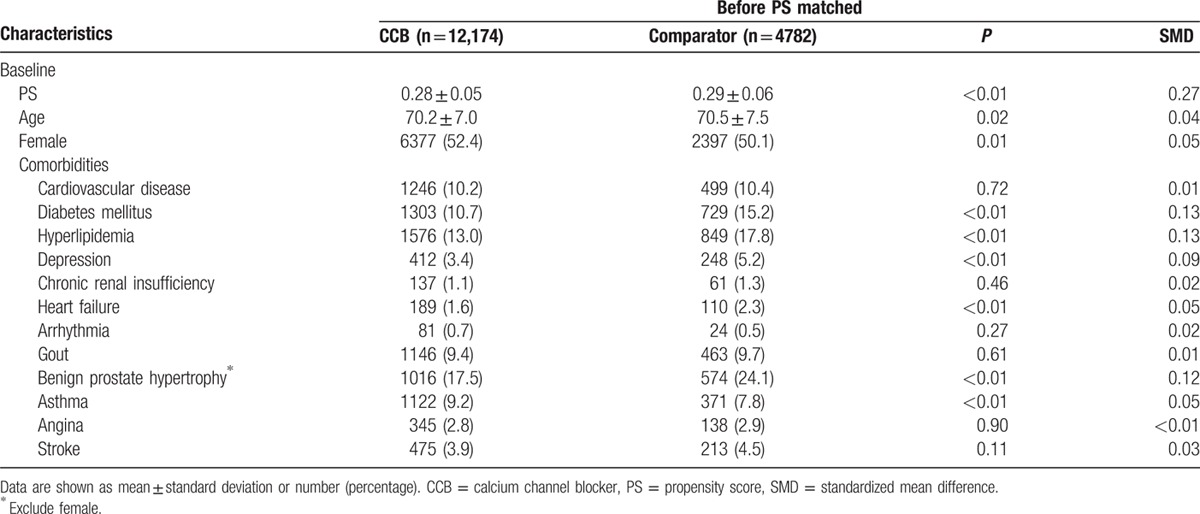
Baseline characteristics between calcium channel blocker exposure group and comparator group.

**Table 2:**
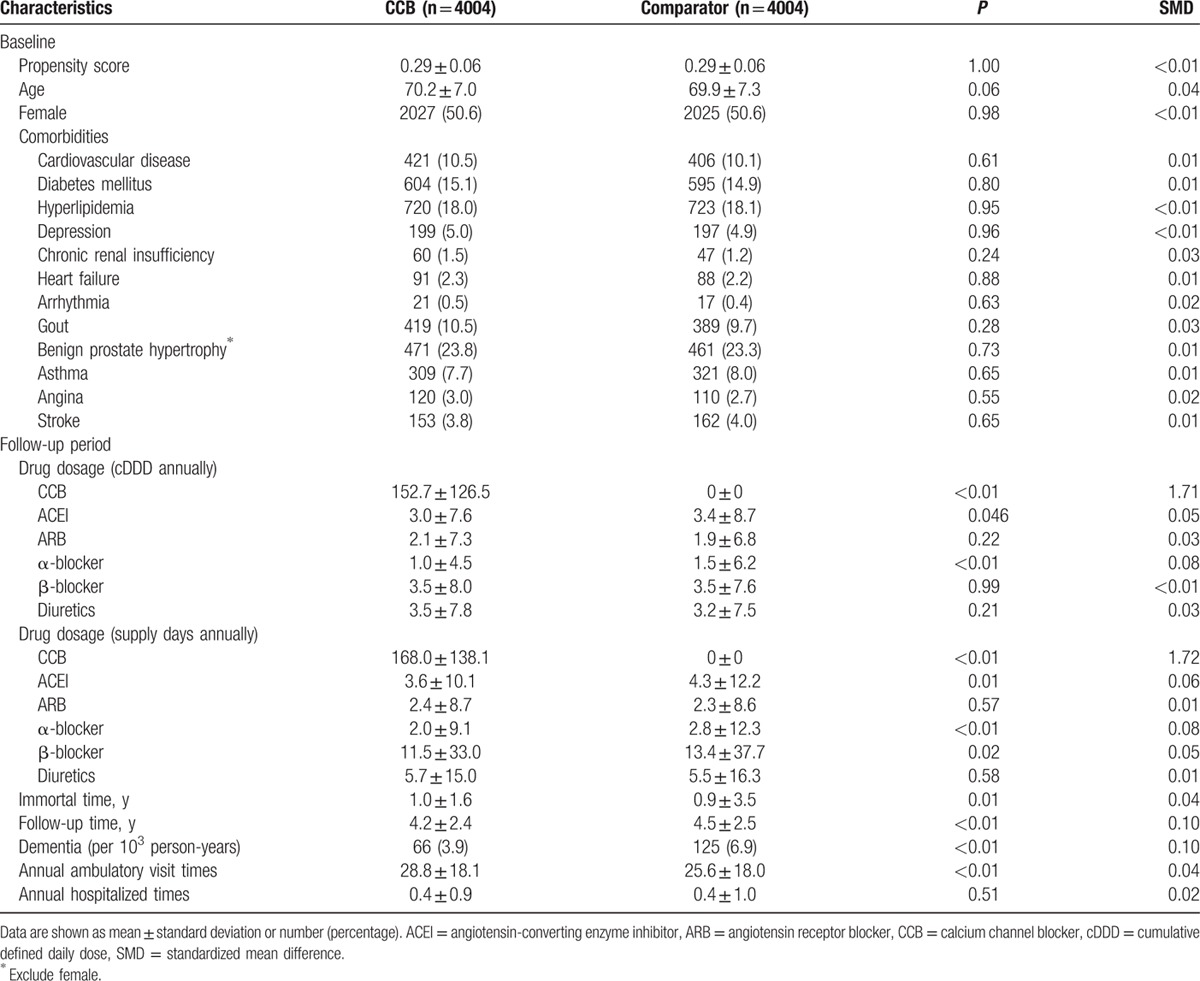
Characteristics at baseline and during follow-up among propensity score matched calcium channel blocker exposure and comparator group.

**Figure 2:**
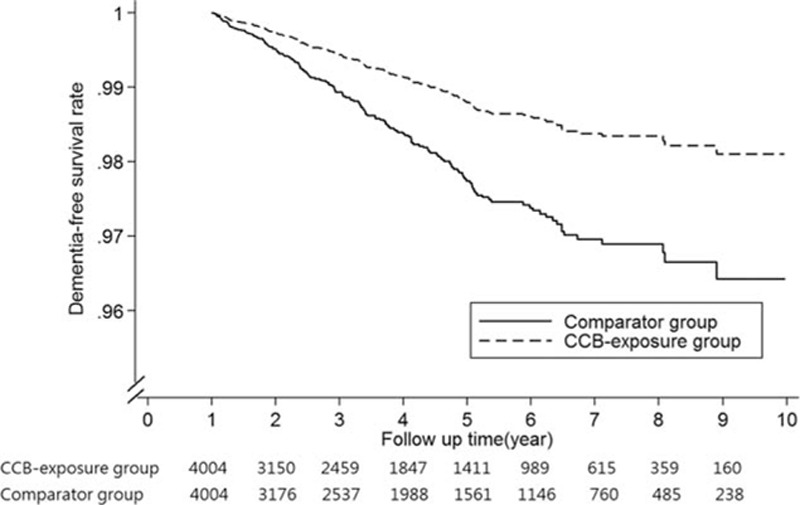
Adjusted dementia-free survival curves of for calcium channel blocker exposure and comparator groups based on robust Cox regression model. Covariates included age, sex, comorbidities, the year of hypertension diagnosis, annual ambulatory visit times, annual hospitalized times, and cumulative defined daily dose of other antihypertensive medications. Follow-up started 1 year after the index date. CCB = calcium channel blocker.

**Figure 3:**
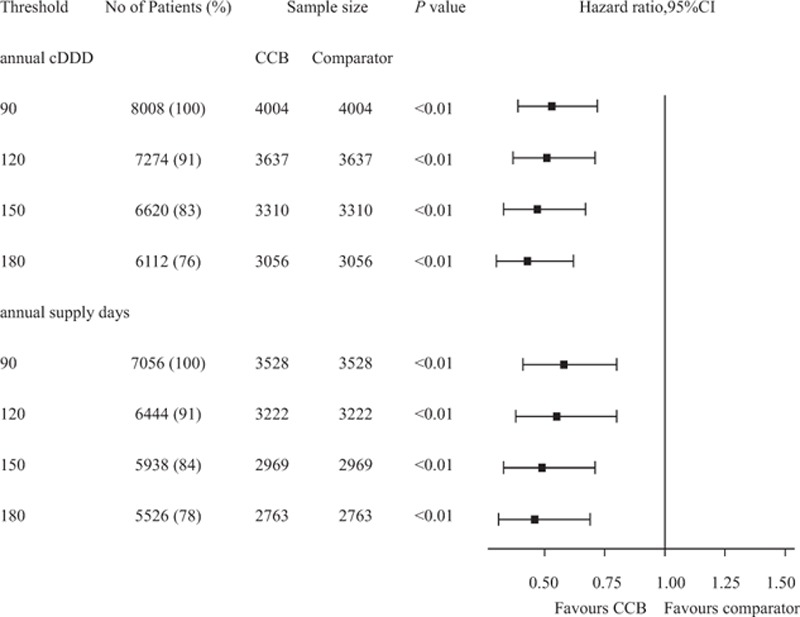
Hazard ratio and 95% confidence interval of dementia risk at various thresholds for calcium channel blocker dosage and supply days in elderly hypertensive patients by robust Cox regression model. CCB = calcium channel blocker, cDDD = cumulative defined daily dose, CI = confidence interval.

Adjusted Cox proportional hazard dementia-free survival curves were shown in Fig. 2. Hypertensive patients treated with CCB use had a significantly better dementia-free survival rate than nonusers (*P* < 0.01). In 4004 PS-matched pairs, the unadjusted HR for developing dementia in the CCB-exposure group was 0.55 (95% CI: 0.41–0.75, *P* < 0.01) as compared with nonusers by robust Cox model. After adjusting for potential confounding factors, the adjusted HR decreased slightly to 0.53 (95% CI: 0.39–0.72, *P* < 0.01) (Table 3). On the basis of the PS-matched cohort, we performed sensitivity analyses using 2 different definitions of cumulative exposure to CCB as follows: drug dosage and exposure duration (Fig. 3). To evaluate the influence of drug dosage, we elevated the cDDD thresholds to 120, 150, and 180 of CCB use. For exposure duration, the CCB treatment period was defined as the drug supply days at the cutoff values of 90, 120, 150, and 180 days. As the drug dosage increased to 180 cDDD, the estimated HR declined slightly from 0.53 to 0.43 relative to nonusers. Similarly, as the drug supply days increased from 90 to 180 days, the estimated HR declined slightly from 0.58 to 0.46 compared with nonusers. In subgroup analysis, association between CCB exposure and dementia did not differ by sex, age (70–80, >80 years), hyperlipdemia, diabetes mellitus, stroke, and depression (Table 4).

**Table 3:**
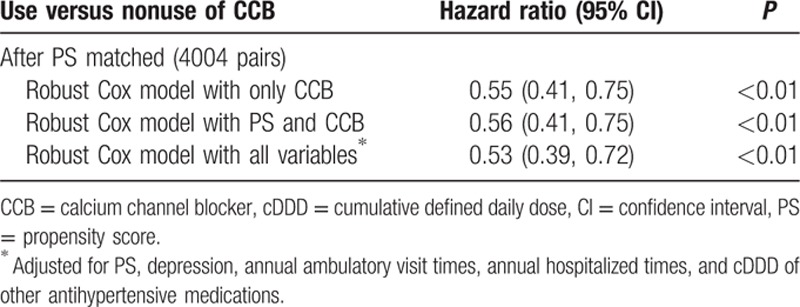
Hazard ratios and 95% confidence intervals for dementia in calcium channel blockers group after propensity score matched.

**Table 4:**
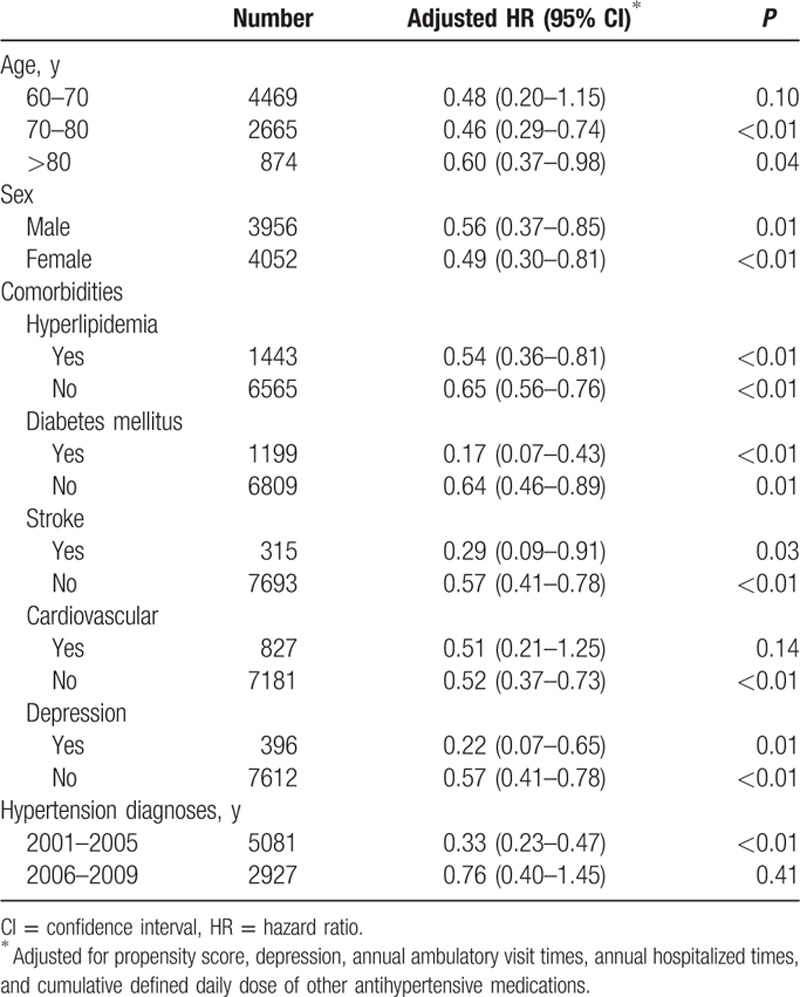
Subgroup analyses for hazard ratio of dementia risk in calcium channel blocker exposure group based on propensity score matched cohort.

## 4 Discussion

There is an urgent need to reduce the risk of developing dementia in the elderly hypertensive patients that is emphasized by the present findings showing that during a mean follow-up of 4.4 years, the incidence of dementia was significantly lower in the CCB-exposure group than that in the comparator group. Moreover, after patients were matched according to PS, they were quite similar in their demographics, baseline comorbidities, as well as treatment with other antihypertensives. Our results demonstrated that a lower risk of developing dementia in the CCB-exposure group compared with nonusers (HR: 0.53, 95% CI: 0.39–0.72). Additional sensitivity and subgroup analyses produced similar results with regard to thresholds of cDDD, drug days supply, and baseline comorbidities.

Our findings of lower risk of dementia for elderly hypertensive patients treated with CCB were consistent with previous randomized double-blind placebo-controlled study, ^[11]^ observation study, ^[12]^ and systematic review. ^[18]^ Another study found that patients using calcium antagonists, compared with those who did not, exhibited higher cognitive function, which was independent of BP levels. ^[25]^ Although the mechanism is unclear, several studies provide explanations of why CCB reduce the risk of developing dementia.[ ^2^ ^14^] First, the preventative effect of CCB on developing dementia is exerted through the inhibition of calcium channel function, in contrast to lowering BP. ^[14]^ Second, in vitro studies showed that CCB attenuated amyloid-beta-induced neuronal decline and prevented cell degeneration, and exerted a neuroprotective effect in animal studies. ^[2]^ Thus, blocking calcium channels would disrupt the pathology of AD. In contrast, other studies found that CCB did not reduce the risk of developing AD.[ ^3^ ^17^] The inconsistent results among previous studies[ ^3^ ^11^ ^12^ ^16^ ^24^] may in part be result from the differences such as study design (e.g., clinical trial, cross-sectional, or longitudinal study), the definition of exposure and comparator groups, the criteria used to assess dementia, the follow-up period, and the characteristics of subjects (e.g., ethnicity and BP). Specifically, we found that longer treatment with CCB as a function of drug dosage and exposure duration decreased the risk of developing dementia. As the threshold dosages increased from 90 to 180, patients who took their drugs more frequently were having slightly lower risk of dementia. The results might indicate that CCB treatment not only lowers high BP but also has a positive effect on preventing dementia. The mechanisms linking the potentially beneficial effect of CCB on preventing dementia remain unclear, and future studies are required to confirm this relationship.

According to international guidelines, ^[1]^ CCB was recommended as the first-line drug to treat hypertension, especially for elderly patients. During study period (2000–2011), the recommendations on how to treat hypertension have not been changed a lot. For prehypertensive patients, lifestyle changes were encouraged for controlling their BP. For patients with BP > 140/90 mm Hg, BP reduction should be initiated by prescribing first-line drugs. We restricted the comparator group as patients treated with no use of CCB and taking each kind of antihypertensives with cDDD < 90. As they were very likely to have mild hypertension as assessed with a mean 4.4-year follow-up and were thus expected to control their BP by alternative strategies (e.g., engaging in a healthier lifestyle) without significant reliance on antihypertensives. This can be explained by the complications related to hypertension during follow-up period in comparator group was similar to that in CCB-exposure group (less than 2 events for cardiovascular disease and ischemic stroke). In Taiwan, combination therapy with at least 2 antihypertensives is common (approximately 56% in 2004). ^[25]^ Thus, we included patients treated with a few other antihypertensives to maintain sufficient sample sizes. In addition, we expected to minimize the effects of other types of antihypertensives used to treat dementia (e.g., ARB) as well as drug interactions. Inevitably, we excluded hypertensive patients who were treated with CCB in combination with other antihypertensives, which limited the generalization of our findings to such patients. Future studies are therefore required to evaluate the potentially positive effects of CCB on elderly patients with consideration of the combination therapy.

The strengths of the present study included utilizing a large population-based claims database (NHIRD), the comprehensive and complete records of prescriptions for antihypertensives, and the implementation of PS matching to avoid confounding bias in acquiring the results. However, potential limitations should be noted. First, although we used the PS-matching method to facilitate a fair comparison among 2 groups, we cannot rule out the effects of unmeasured confounders. For example, data on genetic factors (e.g., polymorphisms of the gene encoding apolipoprotein E [*APOE*]), BP, lifestyle, and education were not available from NHIRD. Fortunately, *APOE* genotypes did not differ between antihypertensive-treated and untreated groups in previous study. ^[16]^ Second, records of BP were not available in NHIRD; thus, it was difficult to compare blood controlling or disease severity among the 2 groups. Although we found that the hypertension-related complications occurred similarly between CCB-exposure and comparator groups during follow-up period, this does not rule out residual confounding. Third, drug dosage of CCB might be overestimated as patients might not have taken their prescribed antihypertensives. When we elevated the threshold to 180 for cDDD or drug days supply, however, the results were similar. Fourth, the misclassification of hypertension and dementia based on claims data may bias the results. As noted above, hypertensive patients were defined as individuals treated with antihypertensive medications. Dementia was diagnosed by a board-qualified certified and well trained neurologist or a psychiatrist. Further, dementia was identified as patients who had catastrophic illness certificates of dementia to minimize the possibility of misclassifying patients. Finally, our findings demonstrated association, but not necessarily causal relationship. Further study is warranted to examine the potential causal effect of CCB use on dementia.

In conclusion, we showed here that the use of CCB for 90 cDDD or more was associated with the lower risk of dementia in the elderly hypertensive patients. The results remain consistent across a wide set of sensitivity and subgroup analyses. Further studies are required to validate potential mechanisms of the positive effects of CCB against the development of dementia.
